# Case Definition for Diagnosed Alzheimer Disease and Related Dementias in Medicare

**DOI:** 10.1001/jamanetworkopen.2024.27610

**Published:** 2024-09-03

**Authors:** Kan Z. Gianattasio, Jason Wachsmuth, Ryan Murphy, Alex Hartzman, Jaleh Montazer, Erin Cutroneo, John Wittenborn, Melinda C. Power, David B. Rein

**Affiliations:** 1 NORC at the University of Chicago, Bethesda, Maryland; 2 Department of Epidemiology, George Washington University School of Public Health, Washington, DC

## Abstract

**Importance:**

Lack of a US dementia surveillance system hinders efforts to support and address disparities among persons living with Alzheimer disease and related dementias (ADRD).

**Objective:**

To review diagnosis and prescription drug code ADRD identification algorithms to develop and implement case definitions for national surveillance.

**Design, Setting, and Participants:**

In this cross-sectional study, a systematic literature review was conducted to identify unique *International Statistical Classification of Diseases, Tenth Revision, Clinical Modification (ICD-10-CM)* and prescription drug codes used by researchers to identify ADRD in administrative records. Code frequency of use, characteristics of beneficiaries identified by codes, and expert and author consensus around code definitions informed code placement into categories indicating highly likely, likely, and possible ADRD. These definitions were applied cross-sectionally to 2017 to 2019 Medicare fee-for-service (FFS) claims and Medicare Advantage (MA) encounter data to classify January 2019 Medicare enrollees. Data analysis was conducted from September 2022 to March 2024.

**Exposures:**

*ICD-10-CM* and national drug codes in FFS claims or MA encounters.

**Main Outcomes and Measures:**

The primary outcome was counts and rates of beneficiaries meeting each case definition. Category-specific age, sex, race and ethnicity, MA enrollment, dual-eligibility, long-term care utilization, mortality, and rural residence distributions, as well as frailty scores and FFS monthly expenditures were also analyzed. Beneficiary characteristics were compared across categories, and age-standardized to minimize confounding by age.

**Results:**

Of the 60 000 869 beneficiaries included (50 853 806 aged 65 years or older [84.8%]; 32 567 891 female [54.3%]; 5 555 571 Hispanic [9.3%]; 6 318 194 non-Hispanic Black [10.5%]; 44 384 980 non-Hispanic White [74.0%]), there were 4 312 496 (7.2%) with highly likely ADRD, 1 124 080 (1.9%) with likely ADRD, and 2 572 176 (4.3%) with possible ADRD, totaling more than 8.0 million with diagnostic evidence of at least possible ADRD. These beneficiaries were older, more frail, more likely to be female, more likely to be dual-eligible, more likely to use long-term care, and more likely to die in 2019 compared with beneficiaries with no evidence of ADRD. These differences became larger when moving from the possible ADRD group to the highly likely ADRD group. Mean (SD) FFS monthly spending was $2966 ($4921) among beneficiaries with highly likely ADRD compared with $936 ($2952) for beneficiaries with no evidence of ADRD. Differences persisted after age standardization.

**Conclusions and Relevance:**

This cross-sectional study of 2019 Medicare beneficiaries identified more than 5.4 million Medicare beneficiaries with evidence of at least likely ADRD in 2019 using the diagnostic case definition. Pending validation against clinical and other methods of ascertainment, this approach can be adopted provisionally for national surveillance.

## Key Points

**Question:**

How many Medicare beneficiaries have diagnostic codes or drug prescriptions indicating Alzheimer disease and related dementias (ADRD) using a refined case definition, and what are the characteristics of these beneficiaries?

**Findings:**

This cross-sectional study of more than 60 million Medicare beneficiaries identified 7.2% with evidence of highly likely ADRD, 1.9% with likely ADRD, and 4.3% with possible ADRD. Beneficiaries with evidence of ADRD were older, more frail, more likely to use long-term care, and more likely to die than those without evidence of ADRD; these differences persisted after age-standardization.

**Meaning:**

In this cross-sectional study, more than 5.4 million Medicare beneficiaries (9.1%) had evidence of likely or highly likely ADRD in 2019; pending validation, this case definition can be adopted provisionally for national surveillance of persons with diagnosed dementia in the Medicare system.

## Teaser

This cross-sectional study uses Medicare claims and encounter data as well as diagnostic and prescription drug codes to develop case definitions for diagnosed Alzheimer disease and related dementias.

## Introduction

Surveillance is a fundamental public health activity. Lack of a US dementia surveillance system hinders public health efforts to support persons living with Alzheimer disease and related dementias (ADRD), address health disparities, and plan ADRD care resources.

Medicare administrative data are an attractive source upon which to build a dementia surveillance system and are commonly used to identify persons living with ADRD, but a consensus diagnostic code case definition does not exist. Perhaps the most widely used definition (the Centers for Medicare and Medicaid Services [CMS] Chronic Conditions Warehouse [CCW] algorithm) uses 22 *International Statistical Classification of Diseases, Tenth Revision, Clinical Modification (ICD-10-CM) *codes, from the commonly accepted G30.X (Alzheimer disease) and F01.XX (vascular dementia), to less specific codes such as R54 (age-related physical debility).^1^ In contrast, most researcher-developed *ICD-10*-*CM*–based algorithms exclude R54, but may include codes such as G31.0 (frontotemporal dementia) that are not in the CCW algorithm.^2,3,4^ Moreover, while some algorithms use Medicare Part D data to identify prescriptions for Alzheimer disease–related drugs,^5,6,7,8^ most do not.

The impact of using different *ICD-10-CM* or prescription codes on the number of people identified or their characteristics is unknown. Because *ICD-10-CM* codes are used for billing (rather than diagnostic) purposes, specific codes may not be sensitive nor specific to dementia, and coding practices may differ systematically by health care practice, patient characteristics, and geography.

We examined how choices of *ICD-10-CM* and prescription drug codes used to identify persons with clinically recognized ADRD in Medicare fee-for-service (FFS) claims and Medicare Advantage (MA) encounter data affect dementia prevalence estimates and characteristics of the people identified. We synthesized this information to develop a new case definition using diagnostic and prescription drug codes that can be applied to administrative data to support surveillance of persons with diagnosed dementia in the Medicare system.

## Methods

This cross-sectional study was deemed exempt from review and the requirement of informed consent by the NORC Institutional Review Board. The reporting of this research follows the Strengthening the Reporting of Observational Studies in Epidemiology (STROBE) reporting guideline.

### *ICD-10-CM* Code Identification

We searched PubMed for articles published from 2012 to 2022, with all-cause dementia or ADRD as a primary exposure or primary outcome, or where the research population of interest was persons living with all-cause dementia or ADRD (eAppendix in Supplement 1). We found 28 studies utilizing 20 distinct researcher-developed *ICD-10-CM* or prescription drug code algorithms in addition to the CCW algorithm (eTable 1 in Supplement 1).^2,3,4,5,6,7,8,9,10,11,12,13,14,15,16,17,18,19,20,21^

We extracted 43 *ICD-10-CM* codes and 5 prescription drugs across algorithms (Table 1 and eTable 1 in Supplement 1). We shared the codes with 3 clinicians (2 neurology clinicians and 1 geriatrics clinician) who provide care to persons living with dementia, who recommended excluding 8 codes deemed to not indicate dementia (Table 1). We grouped the remaining codes into tiers by use frequency (tier 1, ≥15 algorithms; tier 2, 10-14 algorithms; tier 3, 5-9 algorithms; and tier 4, 1-4 algorithms). We designated prescriptions for ADRD-targeting drugs as indicated by National Drug Codes (NDC) without presence of an ADRD *ICD-10-CM* code as tier 5.

**Table 1.** *ICD-10-CM* Codes and Prescription Names Used to Categorize Individuals Across Tiers and ADRD Categories

Tier and *ICD-10-CM* code or prescription drug	Description	Algorithms, No.	ADRD Category
1			
F01.50	Vascular dementia without behavioral disturbance	16	Highly likely ADRD (≥2 codes) or likely ADRD (1 code)
F01.51	Vascular dementia with behavioral disturbance	15	
F01^a^	Vascular dementia	4	
F01.5^a^	Vascular dementia	3	
F02.80	Dementia in other diseases classified elsewhere without behavioral disturbance	18	
F02.81	Dementia in other diseases classified elsewhere with behavioral disturbance	17	
F02^a^	Dementia in other diseases classified elsewhere	4	
F02.8^a^	Dementia in other diseases classified elsewhere	2	
F03.90	Unspecified dementia without behavioral disturbance	17	
F03.91	Unspecified dementia with behavioral disturbance	15	
F03^a^	Unspecified dementia	4	
F03.9^a^	Unspecified dementia	2	
G30.0	Alzheimer disease with early onset	17	
G30.1	Alzheimer disease with late onset	17	
G30.8	Other Alzheimer disease	17	
G30.9	Alzheimer disease, unspecified	19	
G30^a^	Alzheimer disease	7	
2			
G31.01	Pick disease	14	
G31.09	Other frontotemporal neurocognitive disorder	15	
G31.0^a^	Frontotemporal dementia	4	
G31.1	Senile degeneration of brain, not elsewhere classified	14	
G31.83^b^	Dementia with Lewy bodies	11	
R41.81	Age-related cognitive decline	10	
3			
F04	Amnestic disorder due to known physiological condition	7	Possible ADRD
F06.8	Other specified mental disorders due to known physiological condition	6	
G31.2	Degeneration of nervous system due to alcohol	5	
G94	Other disorders of brain in diseases classified elsewhere	8	
4			
F06.1	Catatonic disorder due to known physiological condition	3	
G13.8	Systemic atrophy primarily affecting central nervous system in other diseases classified elsewhere	3	
G31.84^b^	Mild cognitive impairment, so stated	2	
G31.89^b^	Other specified degenerative diseases of nervous system	2	
G31.9	Degenerative disease of nervous system, unspecified	2	
R54	Age-related physical debility	3	
5			
Rivastigmine	NA	4	
Galantamine	NA	4	
Memantine	NA	4	
Donepezil	NA	4	
Tacrine	NA	3	
Not ADRD			
F05	Delirium due to known physiological condition	3	No evidence of ADRD (along with remaining sample)
F06.0	Psychotic disorder with hallucinations due to known physiological condition	2	
F10.27	Alcohol dependence with alcohol-induced persisting dementia	1	
F19.97	Other psychoactive substance use, unspecified with psychoactive substance-induced persisting dementia	1	
G31.81^b^	Alpers disease	1	
G31.82^b^	Leigh disease	1	
G31.85^b^	Corticobasal degeneration	1	
G91.4	Hydrocephalus in diseases classified elsewhere	1	

Abbreviations: ADRD, Alzheimer disease and related dementias; *ICD-10-CM*, *International Statistical Classification of Diseases, Tenth Revision, Clinical Modification*; NA, not applicable.

^a^ Although these codes appear in fewer than 10 algorithms, they are root codes and are categorized alongside their associated detailed codes.

^b^ We dropped root codes G31 and G31.8 (which appeared in 1 algorithm) due to their associated detailed codes being categorized into different tiers.

### Data

We used 100% of the 2017 to 2019 Medicare FFS inpatient, outpatient, carrier, skilled nursing facility, home health agency, and hospice claims; MA inpatient, outpatient, carrier, skilled nursing facility, and home health agency encounter data; and Medicare Part D prescription drug event (PDE) data. We used the minimum dataset (MDS 3.0) to identify long-term care (LTC) utilization. We limited analysis to Medicare beneficiaries with at least Part A (the premium-free Medicare benefit) enrollment in January 2019, nonmissing sex, and a valid US state or territory code based on the Medicare beneficiary summary files. We did not exclude beneficiaries based on age or lack of Part B enrollment because our aim was to identify all people in the Medicare system with evidence of ADRD.

### Statistical Analysis

To categorize beneficiaries with or without evidence of dementia as of 2019, we conducted a cross-sectional analysis of January 2017 to December 2019 FFS and MA data to identify all claims and encounters with a relevant *ICD-10-CM* code listed in any position, and all PDE claims for a relevant NDC. We classified beneficiaries hierarchically, first with a tier 1 *ICD-10-CM* code, then with a tier 2 code among remaining beneficiaries, and so on, identifying only the incremental beneficiaries in each tier if they had not been classified earlier. We compared distributions of age, sex, race and ethnicity (as indicated by the Research Triangle Institute race code^22^), MA enrollment, LTC use, and 2019 mortality across tiers. Race and ethnicity categories included American Indian or Alaska Native, Asian or Pacific Islander, Hispanic, non-Hispanic Black, non-Hispanic White, unknown, and other (defined as any race or ethnicity not otherwise specified); race and ethnicity were included because existing evidence shows that there are disparities in dementia prevalence across race and ethnicity groups. We compared cross-tier beneficiary frailty using a claims-based frailty index (CFI),^23^ an adapted CFI that excludes ADRD codes in tiers 1 to 4, and per-member-per-month (PMPM) spending, averaged across all months of 2019 FFS coverage.

Using these data (eTable 2 in Supplement 1), we found that beneficiaries in tiers 1 and 2 were older, more frail, more likely to be female, in LTC, and die than those in tiers 3 to 5. There were minimal differences in race and ethnicity across tiers, with exception of a higher-than-expected representation of Hispanic and Asian and Pacific Islander beneficiaries in tier 5; however, the overall size of the sample categorized as tier 5 was very small, at just 0.1% (52 338 of 60 000 869 beneficiaries). Based on the findings from the cross-tier comparison and author consensus, we further aggregated codes into 3 categories with decreasing confidence of having a true ADRD diagnosis: a highly likely ADRD category requiring at least 2 claims or encounters on different dates with *ICD-10-CM* codes from tiers 1 or 2; a likely ADRD category requiring 1 claim or encounter with an *ICD-10-10-CM* code from tiers 1 or 2; and a possible ADRD category requiring at least 1 claim or encounter with an *ICD-10-CM* or NDC code from tiers 3, 4, or 5 over a 3-year lookback period. We categorized beneficiaries and reevaluated group demographics, health insurance type, frailty and mortality, and rural residency. We then computed prevalence of highly likely, likely, and possible ADRD within population subgroups defined by these characteristics. We age-standardized to the full analytical population to evaluate differences unconfounded by age.

All analyses were conducted in SAS Enterprise Guide 7.1 and SAS Studio version 3.81 (SAS Institute). Data analysis was conducted from September 2022 to March 2024.

## Results

Of 64 430 729 2019 Medicare beneficiaries, we excluded 3 940 831 due to lack of Part A enrollment in January, 8 due to missing sex, and 489 021 due to a nonvalid US state or territory code, resulting in a total of 60 000 869 beneficiaries (50 853 806 aged 65 years or older [84.8%]; 32 567 891 female [54.3%]; 5 555 571 Hispanic [9.3%]; 6 318 194 non-Hispanic Black [10.5%]; 44 384 980 non-Hispanic White [74.0%]) included in the study sample. Of all beneficiaries, 11 502 479 (19.2%) had Medicaid dual-eligibility, while 23 607 426 (39.3%) had MA. Mean (SD) FFS PMPM spending in 2019 was $1220 ($3426) (Table 2).

**Table 2.** Sample Descriptive Statistics

Variable	Participants, No. (%) (N = 60 000 869)
Sex	
Female	32 567 891 (54.3)
Male	27 432 978 (45.7)
Age, y	
0-44	1 856 066 (3.1)
45-64	7 290 997 (12.2)
65-74	29 878 739 (49.8)
75-84	14 991 100 (25.0)
≥85	5 983 967 (10.0)
Race and ethnicity	
American Indian or Alaska Native	268 476 (0.4)
Asian or Pacific Islander	1 955 343 (3.3)
Hispanic	5 555 571 (9.3)
Non-Hispanic Black	6 318 194 (10.5)
Non-Hispanic White	44 384 980 (74.0)
Other^a^	494 274 (0.8)
Unknown	1 024 031 (1.7)
Health insurance and care utilization	
Dual eligible^b^	11 502 479 (19.2)
Medicare Advantage^c^	23 607 426 (39.3)
Long-term care utilization^d^	937 248 (1.6)
FFS per-member-per-month spending, mean (SD), $^e^	1220 (3426)
Frailty and mortality	
CFI score, mean (SD)^f^	0.169 (0.073)
Adapted CFI score, mean. (SD)^g^	0.164 (0.066)
Decedent	2 285 257 (3.8)
Residence^h^	
Rural	11 744 673 (19.6)
Urban	48 206 558 (80.3)

Abbreviations: CFI, Claims-Based Frailty Index; FFS, fee-for-service.

^a^ Other included any race or ethnicity not otherwise specified.

^b^ Had dual-eligibility for at least 1 month in 2019.

^c^ Enrolled in Medicare Advantage (Medicare Part C) for at least 1 month in 2019.

^d^ Beneficiaries with a long-term care stay qualifying for the Centers for Medicare & Medicaid Services quality measure (≥100 days in the facility without a gap of ≥30 days in between), and which spans January 1, 2019 (identified using the minimum data set).

^e^ Computed using data from all months for which eligible beneficiaries were enrolled in FFS in 2019, including those with partial Medicare Advantage enrollment. Mean monthly FFS spending was computed first at the beneficiary level, and then averaged across all beneficiaries with at least 1 month of FFS enrollment in 2019.

^f^ CFI developed by Kim et al.^23^

^g^ Adapted CFI that excludes *International Statistical Classification of Diseases, Tenth Revision, Clinical Modification (ICD-10-CM)* codes in tiers 1 to 4 (F01.50, F01.51, F02.80, F02.81, F03.90, F03.91, G30.0, G30.1, G30.8, G30.9, G31.01, G31.09, G31.1 G31.83, R41.81, F04, F06.8, G31.2, G94, F06.1, G13.8, G31.84, G31.89, G31.9, and R54).

^h^ Rural and urban do not sum to 100% due to invalid zip codes in claims or zip codes with missing rural-urban commuting area codes.

We identified 4 312 496 beneficiaries (7.2%) as having highly likely ADRD, and 1 124 080 (1.9%) as having likely ADRD (Table 3). The proportion of beneficiaries with highly likely ADRD increased to 8.1% (4 125 639 beneficiaries) after limiting age to 65 years or older, and to 8.8% (4 093 008 beneficiaries) when further limiting to those with both Parts A and B enrollment. The proportion of beneficiaries with likely ADRD increased to 2.1% (996 379 beneficiaries) after these restrictions. Compared with those with likely ADRD, those with highly likely ADRD were older and more frail, more likely to be female and dual-eligible, had over 3 times the rate of LTC utilization (681 923 of 4 312 496 beneficiaries [15.8%] vs 51 332 of 1 124 080 beneficiaries [4.6%]), and almost double the rate of death (828 366 of 4 312 496 beneficiaries [19.2%] vs 129 705 of 1 124 080 beneficiaries [11.5%]). We identified an additional 2 572 176 beneficiaries (4.3%) as having possible ADRD; this percentage increased to 4.8% (2 231 673 beneficiaries) after restricting to beneficiaries aged 65 years or older with Parts A and B enrollment. The possible ADRD group was younger and healthier (lower CFI, mortality, and LTC utilization) than those with highly likely or likely ADRD but was older and less healthy than those with no evidence of ADRD (51 992 117 beneficiaries). Mean (SD) PMPM spending was approximately 3 times as high in the ADRD groups (ranging from $2559 [$2952] among those with possible ADRD to $2966 [$4921] among those with highly likely ADRD) as that of the no ADRD group ($936 [$2952]). Age standardization narrowed differences in sex distribution and death rates, widened differences in race and ethnicity distribution and dual-eligible rates, and had minimal impact on differences in MA enrollment, LTC utilization, and frailty (Figure and eTable 3 in Supplement 1). FFS spending increased slightly for all categories after age standardization.

**Table 3.** Characteristics of Beneficiaries Identified as Having Highly Likely ADRD, Likely ADRD, Possible ADRD, and No Evidence of ADRD

Characteristic	Participants by ADRD likelihood, No. (%) (N = 60 000 869)			
	Highly likely ADRD (n = 4 312 496)	Likely ADRD (n = 1 124 080)	Possible ADRD (n = 2 572 176)	No evidence of ADRD (n = 51 992 117)
Aged 65-117 y^a^^,^^b^	4 125 639 (8.1)	1 013 477 (2.0)	2 245 663 (4.4)	43 448 813 (85.5)
Aged 65-117 y with Medicare Parts A and B^a^^,^^c^	4 093 008 (8.8)	996 379 (2.1)	2 231 673 (4.8)	39 446 099 (84.4)
Sex				
Female	2 719 033 (63.1)	647 070 (57.6)	1 458 310 (56.7)	27 743 478 (53.4)
Male	1 593 463 (36.9)	477 010 (42.4)	1 113 866 (43.3)	24 248 639 (46.6)
Age, y				
0-44	11 009 (0.3)	11 818 (1.1)	50 070 (1.9)	1 783 169 (3.4)
45-64	175 847 (4.1)	98 784 (8.8)	276 443 (10.7)	6 739 923 (13.0)
65-74	770 296 (17.9)	317 226 (28.2)	843 995 (32.8)	27 947 222 (53.8)
75-84	1 615 639 (37.5)	400 267 (35.6)	893 153 (34.7)	12 082 041 (23.2)
≥85	1 739 705 (40.3)	295 985 (26.3)	508 515 (19.8)	3 439 762 (6.6)
Race and ethnicity				
American Indian or Alaska Native	15 764 (0.4)	4880 (0.4)	10 649 (0.4)	237 183 (0.5)
Asian or Pacific Islander	129 497 (3.0)	37 846 (3.4)	64 681 (2.5)	1 723 319 (3.3)
Hispanic	440 359 (10.2)	130 562 (11.6)	222 027 (8.6)	4 762 623 (9.2)
Non-Hispanic Black	493 567 (11.4)	134 455 (12.0)	264 519 (10.3)	5 425 653 (10.4)
Non-Hispanic White	3 183 083 (73.8)	799 467 (71.1)	1 968 048 (76.5)	38 434 382 (73.9)
Other^d^	30 411 (0.7)	8566 (0.8)	18 767 (0.7)	436 530 (0.8)
Unknown	19 815 (0.5)	8304 (0.7)	23 485 (0.9)	972 427 (1.9)
Health insurance and care utilization				
Dual eligible^e^	1 584 782 (36.7)	332 652 (29.6)	638 177 (24.8)	8 946 868 (17.2)
Medicare Advantage (Part C)^f^	1 792 456 (41.6)	503 698 (44.8)	1 119 387 (43.5)	20 191 885 (38.8)
Long-term utilization^g^	681 923 (15.8)	51 332 (4.6)	55 328 (2.2)	148 665 (0.3)
FFS per-member-per-month, mean (SD), $^h^	2966 (4921)	2843 (5429)	2559 (2952)	936 (2952)
Frailty and mortality				
CFI score, mean (SD)^i^	0.294 (0.085)	0.251 (0.088)	0.229 (0.078)	0.154 (0.057)
Adapted CFI score, mean (SD)^j^	0.240 (0.083)	0.223 (0.082)	0.216 (0.077)	0.154 (0.057)
Decedent	828 366 (19.2)	129 705 (11.5)	204 202 (7.9)	1 122 984 (2.2)
Residence^k^				
Rural	781 212 (18.1)	203 647 (18.1)	504 423 (19.6)	10 255 391 (19.7)
Urban	3 529 368 (81.8)	919 831 (81.8)	2 066 793 (80.4)	41 690 566 (80.2)

Abbreviations: ADRD, Alzheimer disease and related dementias; CFI, Claims-Based Frailty Index; FFS, fee-for-service.

^a^ Row percentages (ie, percentage of sample categorized into each ADRD group).

^b^ Sample subset with beneficiary age restricted to age 65 to 117 years (N = 50 833 592).

^c^ Sample subset with beneficiary age restricted to age 65 to 117 years and insurance enrollment restricted to those with both Medicare Part A and Medicare Part B enrollment in January 2019 (N = 46 767 159).

^d^ Other included any race or ethnicity not otherwise specified.

^e^ Had dual-eligibility for at least 1 month in 2019.

^f^ Enrolled in Medicare Advantage (Medicare Part C) for at least 1 month in 2019.

^g^ Beneficiaries with a long-term care stay qualifying for the Centers for Medicare & Medicaid Services quality measure (≥100 days in the facility without a gap of ≥30 days in between), and which spans January 1, 2019 (identified using minimum data set).

^h^ Computed using data from all months for which eligible beneficiaries were enrolled in FFS in 2019, including those with partial Medicare Advantage enrollment. Mean monthly FFS spending was computed first at the beneficiary-level, and then averaged across all beneficiaries with at least 1 month of FFS enrollment in 2019.

^i^ CFI developed by Kim et al.^23^

^j^ Adapted CFI that excludes *International Statistical Classification of Diseases, Tenth Revision, Clinical Modification (ICD-10-CM)* codes in tiers 1 to 4 (F01.50, F01.51, F02.80, F02.81, F03.90, F03.91, G30.0, G30.1, G30.8, G30.9, G31.01, G31.09, G31.1 G31.83, R41.81, F04, F06.8, G31.2, G94, F06.1, G13.8, G31.84, G31.89, G31.9, and R54).

^k^ Rural and urban do not sum to 100% due to invalid zip codes in claims or zip codes with missing rural-urban commuting area codes.

**Figure.**
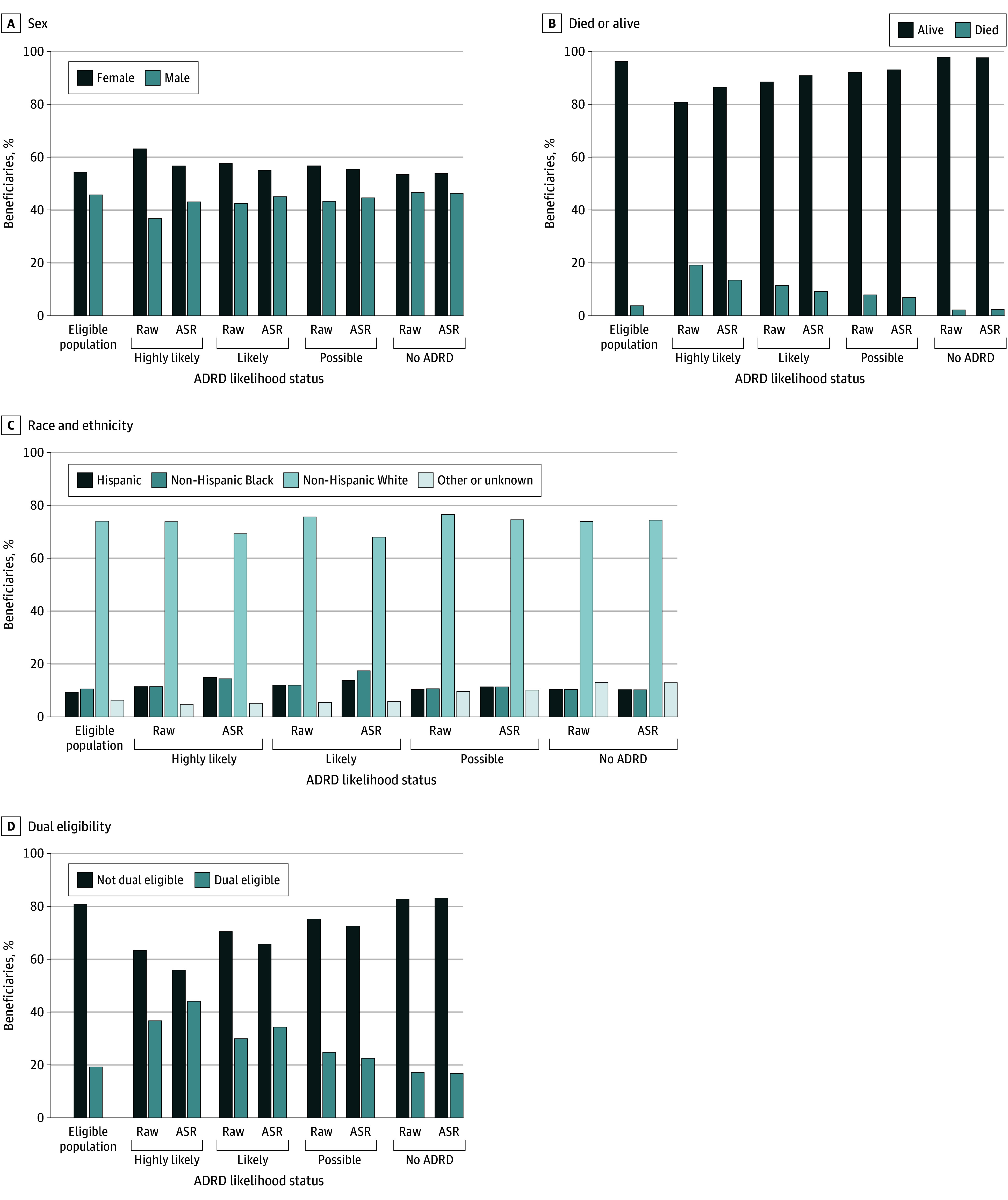
Demographic Distributions Across Alzheimer Disease and Related Dementias (ADRD) Categories (Raw and Age-Standardized) Distributions for sex (A), decedents (B), race and ethnicity (C), and dual-eligibility (D). Beneficiary race and ethnicity was determined using the Research Triangle Institute race code; Other and unknown race and ethnicity category includes Asian and Pacific Islander, American Indian or Alaska Native, and any race or ethnicity not otherwise specified. ASR indicates age-standardized rate.

The proportion of beneficiaries with any evidence of ADRD increased with age, from 6.5% (1 931 517 of 29 878 739 beneficiaries) among beneficiaries aged 65 to 74 years to 42.5% (2 544 205 of 5 983 967) among those aged 85 years or older, with the largest increase seen in the percentage of those with highly likely ADRD (2.6% [770 296 of 29 878 739 beneficiaries] to 29.1% [1 739 705 of 5 983 967 beneficiaries]) (Table 4). Prevalence of any ADRD was higher in females than in males but was similar between non-Hispanic White (5 950 598 beneficiaries [13.4%]), non-Hispanic Black (892 541 beneficiaries [14.1%]), and Hispanic (792 948 beneficiaries [14.3%]) beneficiaries. Those with LTC use were substantially more likely to have ADRD than those with no LTC (681 923 of 937 248 beneficiaries [72.8%] vs 3 630 573 of 59 063 621 beneficiaries [6.1%] categorized as highly likely). Similarly, prevalence of highly likely or likely ADRD was much higher in decedents (958 071 of 2 285 257 beneficiaries [41.9%]) than nondecedents (4 478 505 of 57 715 612 beneficiaries [7.7%]) and in those who were dual-eligible (1 917 434 of 11 502 479 beneficiaries [16.7%]) than among those who were not (3 519 142 of 48 498 390 beneficiaries [7.2%]). MA beneficiaries had a higher prevalence of highly likely or likely ADRD (2 296 154 of 23 607 426 beneficiaries [9.7%]) than FFS beneficiaries (3 140 422 of 36 393 443 beneficiaries [8.6%]), and any evidence of ADRD (4 595 211 of 23 607 426 beneficiaries [14.5%] for MA vs 4 593 211 of 36 393 443 beneficiaries [12.6%] for FFS).

**Table 4.** Prevalence of Highly Likely ADRD, Likely ADRD, and Possible ADRD in Beneficiary Subpopulations Defined by Demographics, Health Care Insurance and Care Utilization, Mortality, and Rural Residency Before and After Standardization to the Age Distribution of the General Population

Variable	Raw distribution of ADRD categorization by beneficiary characteristic, No./total No. (%) (N = 60 000 869)^a^				Age-standardized distribution of ADRD categorization by beneficiary characteristic, %			
	Highly Likely ADRD	Likely ADRD	Possible ADRD	No evidence of ADRD	Highly Likely ADRD	Likely ADRD	Possible ADRD	No evidence of ADRD
Age, y								
0-44	11 009/1 856 066 (0.6)	11 818/1 856 066 (0.6)	50 070/1 856 066 (2.7)	1 783 169/1 856 066 (96.1)	NA	NA	NA	NA
45-64	175 847/7 290 997 (2.4)	98 784/7 290 997 (1.4)	27 6443/7 290 997 (3.8)	6 739 923/7 290 997 (92.4)	NA	NA	NA	NA
65-74	770 296/29 878 739 (2.6)	317 226/29 878 739 (1.1)	843 995/29 878 739 (2.8)	27 947 222/29 878 739 (93.5)	NA	NA	NA	NA
75-84	1 615 639/14 991 100 (10.8)	400 267/14 991 100 (2.7)	893 153/14 991 100 (6.0)	12 082 041/14 991 100 (80.6)	NA	NA	NA	NA
≥85	1 739 705/5 983 967 (29.1)	295 985/5 983 967 (4.9)	508 515/5 983 967 (8.5)	3 439 762/5 983 967 (57.5)	NA	NA	NA	NA
Sex								
Female	2 719 033/32 567 891 (8.3)	647 070/32 567 891 (2)	1 458 310/32 567 891 (4.5)	27 743 478/32 567 891 (85.2)	7.7	1.9	4.4	86.0
Male	1 593 463/27 432 978 (5.8)	477 010/27 432 978 (1.7)	1 113 866/27 432 978 (4.1)	24 248 639/27 432 978 (88.4)	6.4	1.8	4.2	87.6
Race and ethnicity								
Hispanic	440 359/5 555 571 (7.9)	130 562/5 555 571 (2.4)	222 027/5 555 571 (4)	4762623/5555571 (85.7)	8.7	2.5	4.1	84.7
Non-Hispanic Black	493 567/6 318 194 (7.8)	134 455/6 318 194 (2.1)	264 519/6 318 194 (4.2)	5 425 653/6 318 194 (85.9)	9.6	2.4	4.5	83.5
Non-Hispanic White	3 183 083/44 384 980 (7.2)	799 467/44 384 980 (1.8)	1 968 048/44 384 980 (4.4)	38 434 382/44 384 980 (86.6)	6.8	1.7	4.3	87.1
Other^b^	195 487/3 742 124 (5.2)	59 596/3 742 124 (1.6)	117 582/3 742 124 (3.1)	3 369 459/3 742 124 (90.0)	6.4	1.8	3.5	88.3
Health Insurance and care utilization								
Medicare-Medicaid dual eligibility^c^								
Dual eligible	1 584 782/11 502 479 (13.8)	332 652/11 502 479 (2.9)	638 177/11 502 479 (5.5)	8946868/11502479 (77.8)	16.6	3.4	6.0	74.0
Nondual	2 727 714/48 498 390 (5.6)	791 428/48 498 390 (1.6)	1 933 999/48 498 390 (4.0)	43 045 249/48 498 390 (88.8)	5.5	1.6	3.9	89.0
Medicare enrollment^d^								
Fee-for-service	2 520 040/36 393 443 (6.9)	620 382/36 393 443 (1.7)	1 452 789/36 393 443 (4)	31 800 232/36 393 443 (87.4)	7.0	1.7	4.0	87.3
Medicare Advantage (Part C)	1 792 456/23 607 426 (7.6)	503 698/23 607 426 (2.1)	1 119 387/23 607 426 (4.7)	20 191 885/23 607 426 (85.5)	7.5	2.1	4.7	85.6
LTC utilization^e^								
No LTC	3 630 573/59 063 621 (6.1)	1 072 748/59 063 621 (1.8)	2 516 848/59 063 621 (4.3)	51 843 452/59 063 621 (87.8)	6.3	1.8	4.3	87.6
LTC	681 923/937 248 (72.8)	51 332/937 248 (5.5)	55 328/937 248 (5.9)	148 665/937 248 (15.9)	62.8	6.7	7.8	22.7
Mortality								
Did not die in 2019	3 484 130/57 715 612 (6.0)	994 375/57 715 612 (1.7)	2 367 974/57 715 612 (4.1)	50 869 133/57 715 612 (88.1)	6.3	1.8	4.2	87.8
Died in 2019	828 366/2 285 257 (36.2)	129 705/2 285 257 (5.7)	204 202/2 285 257 (8.9)	1 122 984/2 285 257 (49.1)	23.8	4.8	9.2	62.3
Residence rurality								
Rural	781 212/11 744 673 (6.7)	203 647/11 744 673 (1.7)	504 423/11 744 673 (4.3)	10 255 391/11 744 673 (87.3)	6.9	1.8	4.3	87.1
Urban	3 529 368/48 206 558 (7.3)	919 831/48 206 558 (1.9)	2 066 793/48 206 558 (4.3)	41 690 566/48 206 558 (86.5)	7.3	1.9	4.3	86.5

Abbreviations: ADRD, Alzheimer disease and related dementias; LTC, long-term care; NA, not applicable.

^a^ Prevalence of highly likely ADRD, likely ADRD, possible ADRD, and no evidence of ADRD within population subgroups. Percentages are by row.

^b^ Other includes Asian or Pacific Islander, American Indian or Alaska Native, any race or ethnicity not otherwise specified, or unknown.

^c^ Had dual-eligibility for at least 1 month in 2019.

^d^ Enrolled in Medicare Advantage (Medicare Part C) for at least 1 month in 2019.

^e^ Beneficiaries with an LTC stay qualifying for the Centers for Medicare & Medicaid Services quality measure (≥100 days in the facility without a gap of ≥30 days in between), and which spans January 1, 2019 (identified using minimum data set).

Age-standardizing subgroups to the age distribution of the Medicare population resulted in changes in ADRD prevalence estimates in some groups (Table 4). Relative differences in ADRD prevalence narrowed across sex but widened across race and ethnicity groups. Most notably, non-Hispanic White beneficiaries became less likely to have any evidence of ADRD (12.9% across categories), while racial and ethnic minority groups became more likely to have evidence of ADRD (non-Hispanic Black beneficiaries, 16.5%; Hispanic beneficiaries, 15.3%). Among non-Hispanic Black beneficiaries, age standardization resulted in a substantial increase in the proportion of those with highly likely or likely ADRD (9.9% to 12.0%). Age standardization also reduced ADRD prevalence among LTC users (from 72.8% to 62.8% with highly likely ADRD) and decedents (from 36.2% to 23.8% with highly likely ADRD) but had minimal impact in ADRD prevalence among non–LTC users and nondecedents; this is because LTC-users and decedent groups were heavily skewed toward older ages, while the age distribution of the non–LTC users and nondecedent groups mimicked that of the general Medicare population (eTable 4 in Supplement 1).

## Discussion

Among 2019 Medicare beneficiaries in this cross-sectional study, we identified approximately 4.3 million (7.2%) with highly likely ADRD, 1.1 million (1.9%) with likely ADRD, and 2.6 million (4.3%) with possible ADRD, for a total of more than 8.0 million (13.4%) in any category. Specifically, we developed new diagnosis and NDC code ADRD case definitions informed by a systematic review of previous algorithms, author and expert input, and analyses of Medicare data. The review identified 43 *ICD-10-CM* codes and 5 prescription drugs used by the CCW and 20 researcher-developed algorithms to identify ADRD in Medicare data. We divided codes into categories that were likely to indicate ADRD vs those that were possibly ADRD based on past frequency of use by other researchers, characteristics of beneficiaries identified by codes, and author and expert consensus around code definitions. We then added a highly likely category to describe beneficiaries who received 2 or more likely codes on different dates of service. We posit that these categories are superior to previous definitions for provisional use in surveillance systems, but caution that validation is necessary. To our knowledge, this is the first application of claims identification algorithms to all-age FFS and MA beneficiaries. We have used this case definition to compute provisional national-, state-, and county-level estimates of ADRD prevalence and incidence in 2020 Medicare and published them on our dementia surveillance website.^24^ Estimates will be refined pending validation and updated with additional years of data as they become available.

Our 3-level case definition is novel in that it was driven by researcher-consensus as well as data analysis and identifies dementia with varying degrees of certainty. Of note, *ICD-10-CM* codes used to identify possible ADRD have lower researcher consensus and less specific code descriptions (ie, do not contain *dementia *or *Alzheimer*). Use of the possible ADRD codes may reflect physician uncertainty about a dementia diagnosis or medical events involving ADRD-like symptoms in patients without underlying dementia.^25,26,27^ Our definition also excludes several previously used codes that were determined to not indicate ADRD by expert clinicians. Compared with the commonly used CCW algorithm, which similarly uses a 3-year look-back period, our case definition is more specific when limited to the highly likely and likely categories, but broader when also including the possible ADRD category. The CCW algorithm estimated prevalence of 10.7% in 2019 Medicare FFS beneficiaries^28^ falls between our estimates for FFS beneficiaries of 8.6% for highly likely or likely ADRD and 12.6% for all 3 categories.

Importantly, we saw expected and meaningful differences between beneficiaries identified in each ADRD category. Moving from the no evidence of ADRD to the highly likely ADRD groups, beneficiaries became progressively older and more frail and had greater rates of dual-eligibility, LTC use, and death, which is consistent with prior research.^29,30,31,32,33,34,35^ Notably, prevalence of highly likely ADRD was 29.1% in beneficiaries aged 85 years or older, 72.8% in LTC users, and 36.2% in decedents, compared with 7.2% in the general Medicare population. Higher rates of dual-eligibility in ADRD groups may be driven by ADRD beneficiaries spending down assets to qualify for Medicaid and obtain LTC coverage. These differences persisted after age standardization and lend confidence to our case definitions.

Application of our case definitions also showed disparities in diagnosis rates by race in the expected direction—higher dementia risk among non-Hispanic Black beneficiaries relative to non-Hispanic White beneficiaries^36,37^—after age standardization to account for lower life expectancy among non-Hispanic Black individuals.^38^ However, because non-Hispanic Black individuals also have a greater risk of under-diagnosis of ADRD than non-Hispanic White individuals,^39^ disparities in true underlying rates may be higher than observed. Additionally, we found higher-than-expected representation of Hispanic and Asian and Pacific Islander beneficiaries among those that had an ADRD-targeting drug without diagnostic (*ICD-10-CM*) evidence. We hypothesize that differences in cultural perceptions around dementia and cognitive decline (eg, memory loss as a normal aging process)^40,41^ may result in lower utilization of diagnosis codes when providers suspect dementia. Using PDE claims may result in higher and more accurate rates of ADRD among Hispanic and Asian and Pacific Islander individuals despite the overall small number of beneficiaries identified by PDE claims alone.

Finally, also consistent with past research,^29,35,42^ PMPM FFS spending was substantially higher for beneficiaries with evidence of ADRD compared with those with no evidence of ADRD. Medicare FFS PMPM spending was relatively similar across the highly likely, likely, and possible ADRD groups despite differences in frailty and mortality. Medicare FFS spending may not be generalizable to those with MA (for whom costs cannot be computed) and is only part of the economic story. Medicaid is the primary US payer of LTC; higher rates of dual-eligibility and LTC use among the highly likely ADRD group indicate that differences in total federal and state spending between the highly likely ADRD and other groups are likely larger. We also did not capture patient and family health–related out-of-pocket expenses and informal care costs ($203 117 in families caring for a patient living with dementia vs $102 955 in families caring for a patient without dementia over the last 7 years of the patient’s life^42^), forgone wages, or other impacts on informal caregivers, and payments made by other assistance programs. Finally, we caution that our spending measure represents total Medicare FFS spending, rather than the incremental ADRD costs.

### Limitations

This study is limited by at least the following. First, our ADRD case definition was driven by researcher-consensus, and validation against other dementia ascertainment methods (including ascertainment based on in-person clinical and neuropsychological assessments) is necessary. Both over- and under-diagnosis of ADRD have been documented in Medicare claims,^35,39^ and the 8.0 million beneficiaries identified as having some evidence of ADRD by our case definition will include some without ADRD, especially those in the possible category. Similarly, this method only captures documented cases of dementia in Medicare administrative records and cannot capture beneficiaries with unrecognized and/or undocumented ADRD. If we assume a 60% rate of undetected dementia in the US^43^ our estimates would suggest an additional 12 million beneficiaries may be living with ADRD. Additionally, our data show a marginally higher rate of ADRD in MA than in FFS enrollees (14.5% vs 12.6% across the 3 categories), which may reflect beneficiary selection in MA plans, MA vs FFS differences in clinical ADRD assessment and diagnosis rates, differences in claims or encounter documentation, or a combination thereof. Given the rapid rise in MA participation (from 33% in 2017 to 51% in 2023) and variation in MA penetration across counties,^44,45^ it is also important to understand potential differences in performance of this case definition between MA and FFS beneficiaries. As such, validation of this case definition against in-person clinical and other ascertainment methods to assess performance (including sensitivity, specificity, positive predictive value, and negative predictive value), separately for Medicare FFS and MA, is critical for refining and calibrating estimates to accurately capture the diagnosed prevalence and incidence of dementia. Pending validation, our case definitions should be considered provisional. Notably, we expect the possible ADRD category to identify a higher proportion of individuals who do not have ADRD. Thus, it is important to report the possible ADRD category separately from the likely and highly likely ADRD categories in research and surveillance efforts using these case definitions.

Second, evidence for ADRD documented in electronic health or insurance records outside the Medicare system is not captured by our method; this is particularly problematic for beneficiaries without Medicare Parts B or D (7.5% and 25.6% of Medicare enrollees, respectively^43,46^). Third, we deliberately used data from 2017 to 2019 to avoid the COVID-19 pandemic years, which resulted in secular shocks, including excess senior deaths, forgone or deferred care, and increased telehealth, which may have impacted dementia diagnosis. Research is necessary to understand these effects but will necessarily be delayed pending new data. Fourth, Namzaric, a memantine and donepezil combination drug approved in 2014, was not included by any prescription-drug based identification strategy; while the impact of including this drug necessitates further investigation, we anticipate a negligible effect given that just 0.1% of the sample had an ADRD-targeting prescription drug without *ICD-10-CM* evidence. Similarly, ICD-10*-CM* code updates from October 2022 added 29 highly specific codes each under code roots F01 (vascular dementia) F02 (dementia in other diseases classified elsewhere), and F03 (unspecified dementia) (eTable 5 in Supplement 1).^47^ We recommend that applications of our approach to Medicare records beginning in October 2022 include these for identifying highly likely and likely ADRD. Fifth, in developing our case definitions, we only considered use of *ICD-10-CM *codes and prescription drugs but did not consider other criteria of existing ADRD-identification algorithms, including look-back period, types of claims or encounter data considered, number of claims or encounters with relevant *ICD-10-CM * codes required, and time elapsed between claims and encounters; sensitivity analyses around these different criteria are beyond the scope of this paper.

## Conclusions

In this cross-sectional study, our novel case definition for ADRD identified approximately 5.4 million Medicare beneficiaries with evidence of at least likely ADRD in 2019. Pending validation against in-person clinical and other ascertainment methods, this definition can be adopted for provisional use in national surveillance efforts.

## Notes

Supplement 1. **eAppendix.** Literature Review Protocol **eTable 1.** *ICD-10-CM* Codes and Prescription Drugs Used in the CCW and 21 Unique Researcher-Developed Claims-Based Dementia Identification Algorithms **eTable 2.** Characteristics of Beneficiaries Categorized Into Each Tier of *ICD-10-CM *Codes (as Defined by Frequency of Use Across the CCW and Researcher-Developed Algorithms) and NDCs **eTable 3**. Raw and Age-Adjusted Characteristics of Beneficiaries Identified as Having Highly Likely ADRD, Likely ADRD, Possible ADRD, and No Evidence of ADRD **eTable 4.** Beneficiary Age Distribution in the Full Sample, Within LTC Users and Non-Users, and Within Decedents and Non-Decedents **eTable 5.** New Subcodes Associated With F01, F02, and F03 That Went Into Effect in October 2022
eReferences

Supplement 2.
Data Sharing Statement
