# Supplementary material for: Case Definition for Diagnosed Alzheimer Disease and Related Dementias in Medicare
Source: JAMA Netw Open. 2024 Sep 3;7(9):e2427610. doi: 10.1001/jamanetworkopen.2024.27610 (PMC11372506; doi:10.1001/jamanetworkopen.2024.27610)
Supplement: Supplement 1. — eAppendix. Literature Review Protocol eTable 1. ICD-10-CM Codes and Prescription Drugs Used in the CCW and 21 Unique Researcher-Developed Claims-Based Dementia Identification Algorithms eTable 2. Characteristics of Beneficiaries Categorized Into Each Tier of ICD-10-CM Codes (as Defined by Frequency of Use Across the CCW and Researcher-Developed Algorithms) and NDCs eTable 3. Raw and Age-Adjusted Characteristics of Beneficiaries Identified as Having Highly Likely ADRD, Likely ADRD, Possible ADRD, and No Evidence of ADRD eTable 4. Beneficiary Age Distribution in the Full Sample, Within LTC Users and Non-Users, and Within Decedents and Non-Decedents eTable 5. New Subcodes Associated With F01, F02, and F03 That Went Into Effect in October 2022 eReferences [file jamanetwopen-e2427610-s001.pdf]

Supplemental Online Content

Gianattasio KZ, Wachsmith J, Murphy R, et al. Case definition for diagnosed Alzheimer disease and related dementias in Medicare. *JAMA Netw. Open*. 2024;7(8):e2427610. doi:10.1001/jamanetworkopen.2024.27610

eAppendix. Literature Review Protocol

eTable 1. *ICD-10-CM* Codes and Prescription Drugs Used in the CCW and 21 Unique Researcher-Developed Claims-Based Dementia Identification Algorithms

eTable 2. Characteristics of Beneficiaries Categorized Into Each Tier of *ICD-10-CM* Codes (as Defined by Frequency of Use Across the CCW and Researcher-Developed Algorithms) and NDCs

eTable 3. Raw and Age-Adjusted Characteristics of Beneficiaries Identified as Having Highly Likely ADRD, Likely ADRD, Possible ADRD, and No Evidence of ADRD

eTable 4. Beneficiary Age Distribution in the Full Sample, Within LTC Users and Non-Users, and Within Decedents and Non-Decedents

eTable 5. New Subcodes Associated With F01, F02, and F03 That Went Into Effect in October 2022

eReferences

This supplemental material has been provided by the authors to give readers additional information about their work.

© 2024 Gianattasio KZ et al. *JAMA Network Open.*

eAppendix. Literature Review Protocol

We conducted two searches in PubMed for literature published since January 2012:

- On September 20, 2022, we conducted a search using: *"Alzheimer's Disease" AND ("Claims Data" OR "Medicare Claims Data" OR "Diagnostic Incidence" OR "Diagnostic Prevalence" OR "Sensitivity and Specificity" OR "Case Definition Algorithms")*, which yielded 1,680 results after deduplication.
- On December 2, 2022, we conducted a search using: *"Dementia" AND ("Claims Data" OR "Medicare Claims Data" OR "Diagnostic Incidence" OR "Diagnostic Prevalence" OR "Sensitivity and Specificity" OR "Case Definition Algorithms"),* which yielded 1,390 results after de-duplication.

A total of 3,070 de-duplicated articles underwent title/abstract reviews, conducted by one of two reviewers (JM, EC), of which 2,949 were deemed irrelevant. JM/ED then conducted full-text reviews of the remaining articles to identify original research articles with all-cause dementia or ADRD as a primary exposure or primary outcome, or where the research population of interest is persons living with all-cause dementia or ADRD. KZG conducted a second-round review of shortlisted articles. Because our purpose was to identify code algorithms used or developed by the research community, we did not consider study criteria related to methodological rigor and reporting of results that are typically assessed in systematic reviews, and instead used the below inclusion/exclusion criteria:

Inclusion Criteria:

- Original research articles published in English since January 1 2012
- Used Medicare claims/encounter data *ICD-10-CM* codes and/or part D NDC codes to identify individuals with dementia
- Provides list of *ICD-10-CM* code and/or prescription drug names used to identify dementia
- All-cause ADRD or dementia is the primary exposure, primary outcome, or patient population of interest; or where the goal of the study is to develop/validate all-cause dementia or ADRD *ICD-10-CM*/NDC code algorithm

Exclusion Criteria:

- Studies limited to specific dementia subtypes
- Studies including conditions outside dementia (e.g. Parkinson's disease, mild cognitive impairment (MCI), hallucinations)
- Studies using additional sources of information (e.g. electronic health records, physician assessment tools) to identify dementia

A total of 29 met our inclusion/exclusion criteria, yielding 20 distinct researcher-developed *ICD-10-CM*/ NDC-based algorithms, in addition to the CCW algorithm, for identifying all-cause dementia.[1–20]

© 2024 Gianattasio KZ et al. *JAMA Network Open.*

eTable 1. *ICD-10-CM* Codes and Prescription Drugs Used in the CCW and 21 Unique Researcher-Developed Claims-Based Dementia Identification Algorithms

**Code**	**Number of algorithms**	**CCW**	**Gianattasio 2022**	**Jain 2020**	**Borson 2021**	**Grodstein 2021**	**Moura 2021**	**Powell 2022**	**Liu 2021**	**Du 2022**	**Khalid 2022**	**Thunell 2019**	**Wang 2021**	**Branigan 2020**	**Wiemken 2021**	**Chern 2022**	**Rashid 2022**	**Maust 2021**	**Mahmoudi 2022**	**Hua 2022**	**Sattui 2002**	**Sullivan 2021**
F00 [a]	3								X				X								X	
F01	4								X				X		X						X	
F01.5	3									X					X						X	
F01.50	16	X	X	X	X	X	X		X			X		X	X		X	X	X	X	X	X
F01.51	15	X	X	X	X	X	X		X			X		X	X			X	X	X	X	X
F02	4								X		X		X		X							
F02.8	2										X				X							
F02.80	18	X	X	X	X	X	X		X	X	X	X		X	X	X	X	X	X	X		X
F02.81	17	X	X	X	X	X	X		X	X	X	X		X	X	X		X	X	X		X
F03	4								X				X		X						X	
F03.9	2														X						X	
F03.90	17	X	X	X	X	X	X		X	X		X		X	X		X	X	X	X	X	X
F03.91	15	X	X	X	X	X			X	X		X		X	X			X	X	X	X	X
F04	7	X	X	X		X			X			X						X				
F05	3	X							X													X
F05.1 [a]	1								X													
F06.0	2		X							X												
F06.1	3	X							X									X				
F06.8	6	X	X						X	X		X						X				
F10.27	1									X												

© 2024 Gianattasio KZ et al. *JAMA Network Open.*

Code	Number of algorithms	CCW	Gianattasio 2022	Jain 2020	Borson 2021	Grodstein 2021	Moura 2021	Powell 2022	Liu 2021	Du 2022	Khalid 2022	Thunell 2019	Wang 2021	Branigan 2020	Wiemken 2021	Chern 2022	Rashid 2022	Maust 2021	Mahmoudi 2022	Hua 2022	Sattui 2002	Sullivan 2021
F19.97	1									X												
G13.8	3	X							X			X										
G30	7								X	X	X		X		X	X					X	
G30.0	17	X	X	X	X	X	X		X	X	X	X		X	X		X	X	X	X	X	
G30.1	17	X	X	X	X	X	X		X	X	X	X		X	X		X	X	X	X	X	
G30.8	17	X	X	X	X	X	X		X	X	X	X		X	X		X	X	X	X	X	
G30.9	19	X	X	X	X	X	X		X	X	X	X		X	X	X	X	X	X	X	X	X
G31	1																				X	
G31.0	4		X										X					X			X	
G31.01	14	X	X		X	X	X		X	X		X			X		X	X		X	X	X
G31.09	15	X	X		X	X	X		X	X		X		X	X		X	X		X	X	X
G31.1	14	X	X			X	X		X	X		X			X	X	X	X		X	X	X
G31.2	5	X				X	X		X												X	
G31.8	1																				X	
G31.81	1																				X	
G31.82	1																				X	
G31.83	12		X		X	X			X	X			X	X			X	X		X	X	X
G31.84	2									X											X	
G31.85	1																				X	
G31.89	2									X											X	
G31.9	2									X											X	
G91.4	1						X															

© 2024 Gianattasio KZ et al. *JAMA Network Open.*

Code	Number of algorithms	CCW	Gianattasio 2022	Jain 2020	Borson 2021	Grodstein 2021	Moura 2021	Powell 2022	Liu 2021	Du 2022	Khalid 2022	Thunell 2019	Wang 2021	Branigan 2020	Wiemken 2021	Chern 2022	Rashid 2022	Maust 2021	Mahmoudi 2022	Hua 2022	Sattui 2002	Sullivan 2021
G94	8	X	X						X	X		X						X		X		X
R41.81	10	X	X	X		X	X		X	X		X						X				X
R54	3	X							X			X										
Galantamine	4							X				X					X				X	
Rivastigmine	4							X				X					X				X	
Memantine	4							X				X					X				X	
Donepezil	4							X				X					X				X	
Tacrine	3							X									X				X	

[a] Not *ICD-10-CM* codes accepted for use by CMS, and therefore excluded from our subsequent analyses.

© 2024 Gianattasio KZ et al. *JAMA Network Open.*

eTable 2. Characteristics of Beneficiaries Categorized Into Each Tier of *ICD-10-CM* Codes (as Defined by Frequency of Use Across the CCW and Researcher-Developed Algorithms) and NDCs

Variable	N (%) / Mean (SD)						
	**Tier 1 (15+ algorithms)**	**Tier 2 (10-14 algorithms)**	**Tier 3 (5-9 algorithms)**	**Tier 4 (1-4 algorithms)**	**Tier 5 (ADRD-targeting drug without *ICD-10-CM*)**	**Tier 6 (non-ADRD codes)**	**No evidence of ADRD**
N	5,246,241 (8.7%)	190,335 (0.3%)	125,367 (0.2%)	2,394,471 (4.0%)	52,338 (0.1%)	245,229 (0.4%)	51,746,888 (86.2%)
Female, N (%)	3,253,769 (62.0%)	112,334 (59.0%)	62,625 (50.0%)	1,363,946 (57.0%)	31,739 (60.6%)	119,811 (48.9%)	27,623,667 (53.4%)
**Age**							
00-44, N (%)	21,832 (0.4%)	995 (0.5%)	11,416 (9.1%)	37,449 (1.6%)	1,205 (2.3%)	15,485 (6.3%)	1,767,684 (3.4%)
45-64, N (%)	263,872 (5.0%)	10,759 (5.7%)	32,628 (26.0%)	236,408 (9.9%)	7,407 (14.2%)	51,704 (21.1%)	6,688,219 (12.9%)
65-74, N (%)	1,028,317 (19.6%)	59,205 (31.1%)	45,081 (36.0%)	781,469 (32.6%)	17,445 (33.3%)	81,463 (33.2%)	27,865,759 (53.9%)
75-84, N (%)	1,945,303 (37.1%)	70,603 (37.1%)	27,233 (21.7%)	847,275 (35.4%)	18,645 (35.6%)	64,237 (26.2%)	12,017,804 (23.2%)
85+, N (%)	1,986,917 (37.9%)	48,773 (25.6%)	9,009 (7.2%)	491,870 (20.5%)	7,636 (14.6%)	32,340 (13.2%)	3,407,422 (6.6%)
**Race/ethnicity**							
AIAN, N (%)	19,917 (0.4%)	727 (0.4%)	791 (0.6%)	9,692 (0.4%)	166 (0.3%)	1,443 (0.6%)	235,740 (0.5%)
API, N (%)	161,866 (3.1%)	5,477 (2.9%)	2,459 (2.0%)	58,751 (2.5%)	3,471 (6.6%)	5,319 (2.2%)	1,718,000 (3.3%)
Hispanic, N (%)	544,635 (10.4%)	26,286 (13.8%)	11,050 (8.8%)	201,383 (8.4%)	9,594 (18.3%)	17,232 (7.0%)	4,745,391 (9.2%)
NHB, N (%)	614,356 (11.7%)	13,666 (7.2%)	14,989 (12.0%)	245,127 (10.2%)	4,403 (8.4%)	28,843 (11.8%)	5,396,810 (10.4%)
NHW, N (%)	3,841,252 (73.2%)	141,298 (74.2%)	93,100 (74.3%)	1,841,438 (76.9%)	33,510 (64.0%)	187,995 (76.7%)	38,246,387 (73.9%)
Other, N (%)	37,699 (0.7%)	1,278 (0.7%)	931 (0.7%)	17,339 (0.7%)	497 (0.9%)	1,767 (0.7%)	434,763 (0.8%)
Unknown, N (%)	26,516 (0.5%)	1,603 (0.8%)	2,047 (1.6%)	20,741 (0.9%)	697 (1.3%)	2,630 (1.1%)	969,797 (1.9%)
**Health insurance and care utilization**							
Dual, N (%) [a]	1,876,555 (35.8%)	40,879 (21.5%)	46,724 (37.3%)	569,051 (23.8%)	22,402 (42.8%)	85,996 (35.1%)	8,860,872 (17.1%)
MA, N (%) [b]	2,219,317 (42.3%)	76,837 (40.4%)	45,967 (36.7%)	1,050,823 (43.9%)	22,597 (43.2%)	92,924 (37.9%)	20,098,961 (38.8%)
LTC, N (%) [c]	727,580 (13.9%)	5,675 (3%)	3,678 (2.9%)	51,050 (2.1%)	600 (1.1%)	8,576 (3.5%)	140,089 (0.3%)
FFS PMPM, mean (SD) [d]	$2,961 ($5,022)	$2,411 ($5,053)	$2,685 ($5,484)	$2,582 ($5,190)	$1,135 ($2,745)	$5,798 ($11,630)	$908 ($2,798)
**Frailty and mortality**							
CFI, mean (SD)	0.287 (0.087)	0.226 (0.081)	0.227 (0.082)	0.230 (0.078)	0.191 (0.063)	0.267 (0.091)	0.154 (0.056)
Adapted CFI, mean (SD)	0.237 (0.083)	0.214 (0.079)	0.219 (0.079)	0.216 (0.077)	0.191 (0.062)	0.267 (0.091)	0.154 (0.056)

© 2024 Gianattasio KZ et al. *JAMA Network Open.*

Decedent, N (%)	940,932 (17.9%)	17,139 (9.0%)	7,852 (6.3%)	194,729 (8.1%)	1,621 (3.1%)	44,369 (18.1%)	1,078,615 (2.1%)
**Residence**							
Rural, N (%)	947,847 (18.1%)	37,012 (19.4%)	19,705 (15.7%)	475,254 (19.8%)	9,464 (18.1%)	43,442 (17.7%)	10,211,949 (19.7%)
Urban, N (%)	4,295,963 (81.9%)	153,236 (80.5%)	105,556 (84.2%)	1,918,385 (80.1%)	42,852 (81.9%)	201,721 (82.3%)	41,488,845 (80.2%)

Abbreviations: ADRD = Alzheimer's disease and related dementias; API = Asian and Pacific Islander; AIAN = American Indian and Alaskan Native; CFI = Claims-based frailty index; FFS = fee-for-service; MA = Medicare Advantage (part C); NHB = non-Hispanic Black; NHW = non-Hispanic White; PMPM = per-member per month; SD (standard deviation);

[a] Had dual-eligibility for at least one month in 2019.

[b] Enrolled in Medicare Advantage (Medicare Part C) for at least one month in 2019.

[c] Beneficiaries with a long-term care stay qualifying for the Centers for Medicare & Medicaid Services quality measure (≥100 days in the facility without a gap of ≥30 days in between), and which spans January 1, 2019; identified using minimum data set.

[d] Computed using data from all months for which eligible beneficiaries were enrolled in FFS in 2019, including those with partial MA enrollment. Mean monthly FFS spending is computed first at the beneficiary-level, and then averaged across all beneficiaries with at least 1 month of FFS enrollment in 2019.

[e] CFI developed by Kim et al.[23]

[f] Adapted CFI that excludes *ICD-10-CM* codes in tiers 1-4 (F01.50, F01.51, F02.80, F02.81, F03.90, F03.91, G30.0, G30.1, G30.8, G30.9, G31.01, G31.09, G31.1 G31.83, R41.81, F04, F06.8, G31.2, G94, F06.1, G13.8, G31.84, G31.89, G31.9, and R54).

[g] Rural and urban do not sum to 100% due to invalid zip codes in claims or zip codes with missing RUCA codes .

© 2024 Gianattasio KZ et al. *JAMA Network Open.*

eTable 3. Raw and Age-Adjusted Characteristics of Beneficiaries Identified as Having Highly Likely ADRD, Likely ADRD, Possible ADRD, and No Evidence of ADRD

	Full sample	Highly likely ADRD		Likely ADRD		Possible ADRD		No evidence of ADRD	
		Raw	ASR/ASM	Raw	ASR/ASM	Raw	ASR/ASM	Raw	ASR/ASM
**Demographics**									
Age									
0-44, %	3.1	0.3		1.1		1.9		3.4	
45-64, %	12.2	4.1		8.8		10.7		13.0	
65-74, %	49.8	17.9		28.2		32.8		53.8	
75-84, %	25.0	37.5		35.6		34.7		23.2	
85+, %	10.0	40.3		26.3		19.8		6.6	
Female, %	54.3	63.1	56.9	57.6	55.5	56.7	55.4	53.4	53.7
Race/ethnicity									
Hispanic, %	9.3	10.2	11.4	11.6	12.7	8.6	9.2	9.2	9.1
Non-Hispanic Black, %	10.5	11.4	14.4	12.0	13.7	10.3	11.3	10.4	10.2
Non-Hispanic White, %	74.0	73.8	69.2	71.1	68.0	76.5	74.5	73.9	74.4
Other/unknown, % [a]	6.2	4.6	5.0	5.3	5.7	4.5	5.0	6.5	6.4
**Health insurance and care utilization**									
Dual Eligible, % [b]	19.2	36.7	44.1	29.6	34.3	24.8	27.5	17.2	16.8
MA, % [c]	39.3	41.6	43.2	44.8	46.4	43.5	44.2	38.8	38.9
LTC utilization, % [d]	1.6	15.8	14.7	4.6	4.6	2.2	2.1	0.3	0.3
FFS PMPM, mean [e]	$1,220	$2,966	$3,086	$2,843	$2,915	$2,559	$2,600	$936	$954
**Frailty and mortality**									
CFI, mean [f]	0.169	0.294	0.288	0.251	0.248	0.229	0.228	0.154	0.155
Adapted CFI, mean [g]	0.164	0.24	0.238	0.223	0.221	0.216	0.214	0.154	0.155
Decedent, %	3.8	19.2	13.5	11.5	9.2	7.9	7.0	2.2	2.4
**Residence**									
Rural, %	19.6	18.1	18.5	18.1	18.1	19.6	19.7	19.7	19.7
Urban, %	80.3	81.8	81.4	81.8	81.9	80.4	80.3	80.2	80.2

© 2024 Gianattasio KZ et al. *JAMA Network Open.*

Abbreviations: ADRD = Alzheimer's disease and related dementias; ASM = age-adjustment mean; ASR = age-adjusted rate; CFI = Claims-based frailty index; FFS = fee-for-service; LTC = long-term care; MA = Medicare Advantage (part C); PMPM = per-member per month.

[a] Includes: Asian/Pacific Islander, American Indian/Alaskan Native, Other, Unknown

[b] Had dual-eligibility for at least one month in 2019.

[c] Enrolled in Medicare Advantage (Medicare Part C) for at least one month in 2019.

[d] Beneficiaries with a LTC stay qualifying for the Centers for Medicare & Medicaid Services quality measure (≥100 days in the facility without a gap of ≥30 days in between), and which spans January 1, 2019; identified using minimum data set.

[e] Computed using data from all months for which eligible beneficiaries were enrolled in FFS in 2019, including those with partial MA enrollment. Mean monthly FFS spending is computed first at the beneficiary-level, and then averaged across all beneficiaries with at least 1 month of FFS enrollment in 2019. [f]

[f]CFI developed by Kim et al.[23]

[g] Adapted CFI that excludes *ICD-10-CM* codes in tiers 1-4 (F01.50, F01.51, F02.80, F02.81, F03.90, F03.91, G30.0, G30.1, G30.8, G30.9, G31.01, G31.09, G31.1 G31.83, R41.81, F04, F06.8, G31.2, G94, F06.1, G13.8, G31.84, G31.89, G31.9, and R54).

© 2024 Gianattasio KZ et al. *JAMA Network Open.*

eTable 4. Beneficiary Age Distribution in the Full Sample, Within LTC Users and Non-Users, and Within Decedents and Non-Decedents

Age group	Full Sample	LTC user		Decedent	
		No	Yes	No	Yes
00-44	3.1%	3.1%	0.8%	3.2%	0.8%
45-64	12.2%	12.2%	9.1%	12.3%	8.0%
65-74	49.8%	50.3%	18.9%	50.8%	24.1%
75-84	25.0%	24.9%	28.7%	24.8%	30.5%
85+	10.0%	9.5%	42.4%	8.9%	36.6%

Abbreviations: LTC = long-term care

© 2024 Gianattasio KZ et al. *JAMA Network Open.*

eTable 5. New Subcodes Associated With F01, F02, and F03 That Went Into Effect in October 2022.

Code [a]	New definition	Old definition
F0X [b]	"Dementia"	"Dementia
F0X.X [b]	"Dementia", unspecified severity	"Dementia
F0X.X0 [b]	"Dementia", unspecified severity, without behavioral disturbance, psychotic disturbance, mood disturbance, and anxiety	"Dementia" without behavioral disturbance
F0X.X1 [b]	"Dementia", unspecified severity , with behavioral disturbance	"Dementia" with behavioral disturbance
F0X.X11	"Dementia" unspecified severity, with agitation	
F0X.X18	"Dementia" unspecified severity, with other behavioral disturbance	
F0X.X2	"Dementia" unspecified severity, with psychotic disturbance	
F0X.X3	"Dementia" unspecified severity, with mood disturbance	
F0X.X4	"Dementia" unspecified severity, with anxiety	
F0X.A	"Dementia" mild	
F0X.A0	"Dementia" mild, without behavioral disturbance, psychotic disturbance, mood disturbance, and anxiety	
F0X.A1	"Dementia" mild, with behavioral disturbance	
F0X.A11	"Dementia" mild, with agitation	
F0X.A18	"Dementia" mild, with other behavioral disturbance	
F0X.A2	"Dementia" mild, with psychotic disturbance	
F0X.A3	"Dementia" mild, with mood disturbance	
F0X.A4	"Dementia" mild, with anxiety	
F0X.B	"Dementia" moderate	
F0X.B0	"Dementia" moderate, without behavioral disturbance, psychotic disturbance, mood disturbance, and anxiety	
F0X.B1	"Dementia" moderate, with behavioral disturbance	
F0X.B11	"Dementia" moderate, with agitation	
F0X.B18	"Dementia" moderate, with other behavioral disturbance	
F0X.B2	"Dementia" moderate, with psychotic disturbance	
F0X.B3	"Dementia" moderate, with mood disturbance	
F0X.B4	"Dementia" moderate, with anxiety	
F0X.C	"Dementia" severe	

© 2024 Gianattasio KZ et al. *JAMA Network Open.*

Code [a]	New definition	Old definition
F0X.C0	"Dementia" severe, without behavioral disturbance, psychotic disturbance, mood disturbance, and anxiety	
F0X.C1	"Dementia" severe, with behavioral disturbance	
F0X.C11	"Dementia" severe, with agitation	
F0X.C18	"Dementia" severe, with other behavioral disturbance	
F0X.C2	"Dementia" severe, with psychotic disturbance	
F0X.C3	"Dementia" severe, with mood disturbance	
F0X.C4	"Dementia" severe, with anxiety	

[a] F01/F01.5 = Vascular dementia; F02/F02.8 = Dementia in other diseases classified elsewhere; F03/F03.9 = Unspecified dementia

[b] Existing code with updated definition

© 2024 Gianattasio KZ et al. *JAMA Network Open.*

eReferences

1. Gianattasio KZ, Moghtaderi A, Lupu D, Prather C, Power MC. Evaluation of Federal Policy Changes to the Hospice Benefit and Use of Hospice for Persons With ADRD. *JAMA Health Forum*. 2022;3(5):e220900. doi:10.1001/jamahealthforum.2022.0900

2. Jain S, Rosenbaum PR, Reiter JG, et al. Using Medicare claims in identifying Alzheimer's disease and related dementias. *Alzheimer's & Dementia*. Published online 2022. doi:10.1002/alz.12199.

3. Borson S, Chen A, Wang SE, Nguyen HQ. Patterns of incident dementia codes during the COVID-19 pandemic at an integrated healthcare system. *J Am Geriatr Soc*. 2021;69(12):3389-3396. doi:10.1111/jgs.17527

4. Grodstein F, Chang CH, Capuano AW, et al. Identification of Dementia in Recent Medicare Claims Data, Compared With Rigorous Clinical Assessments. *The Journals of Gerontology: Series A*. Published online December 17, 2021. doi:10.1093/gerona/glab377

5. Moura LMVR, Festa N, Price M, et al. Identifying Medicare beneficiaries with dementia. *J Am Geriatr Soc*. 2021;69(8):2240-2251. doi:10.1111/jgs.17183

6. Powell DS, Brenowitz WD, Yaffe K, et al. Examining the Combined Estimated Effects of Hearing Loss and Depressive Symptoms on Risk of Cognitive Decline and Incident Dementia. *The Journals of Gerontology: Series B*. 2022;77(5):839-849. doi:10.1093/geronb/gbab194

7. Liu B, Ornstein KA, Alpert N, et al. Trends of hospitalizations among patients with both cancer and dementia diagnoses in New York 2007-2017. *Healthcare*. 2021;9(3). doi:10.1016/j.hjdsi.2021.100565

8. Du XL, Simpson LM, Osani MC, Yama JM, Davis BR. Risk of Developing Alzheimer's Disease and Related Dementias in ALLHAT Trial Participants Receiving Diuretic, ACE-Inhibitor, or Calcium-Channel Blocker with 18 Years of Follow-Up. *J Alzheimers Dis Parkinsonism*. 2022;12(3):1-28.

9. Thunell J, Ferido P, Zissimopoulos J. Measuring Alzheimer's Disease and Other Dementias in Diverse Populations Using Medicare Claims Data. *Journal of Alzheimer's Disease*. 2019;72(1):29-33. doi:10.3233/JAD-190310

10. Branigan GL, Soto M, Neumayer L, Rodgers K, Brinton RD. Association Between Hormone-Modulating Breast Cancer Therapies and Incidence of Neurodegenerative Outcomes for Women With Breast Cancer. *JAMA Netw Open*. 2020;3(3):e201541. doi:10.1001/jamanetworkopen.2020.1541

11. Wiemken TL, Salas J, Morley JE, Hoft DF, Jacobs C, Scherrer JF. Comparison of rates of dementia among older adult recipients of two, one, or no vaccinations. *J Am Geriatr Soc*. 2022;70(4):1157-1168. doi:10.1111/jgs.17606

12. Chern A, Sharma RK, Golub JS. Hearing Loss and Incident Dementia: Claims Data from the New York SPARCS Database. *Otology and Neurotology*. 2022;43(1):36-41. doi:10.1097/MAO.0000000000003338

© 2024 Gianattasio KZ et al. *JAMA Network Open.*

13. Rashid N, Wetmore JB, Irfan M, Peng Y, Abler V. Medicare claims analysis of agents used to manage dementia-related psychosis: A treatment pattern study. *Int Clin Psychopharmacol*. 2022;37(3):84-91. doi:10.1097/YIC.0000000000000401

14. Maust DT, Strominger J, Kim HM, et al. Prevalence of Central Nervous System-Active Polypharmacy among Older Adults with Dementia in the US. *JAMA - Journal of the American Medical Association*. 2021;325(10):952-961. doi:10.1001/jama.2021.1195

15. Mahmoudi E, Lin P, Kamdar N, Gonzales G, Norcott A, Peterson MD. Risk of early- and late-onset Alzheimer disease and related dementia in adults with cerebral palsy. *Dev Med Child Neurol*. 2022;64(3):372-378. doi:10.1111/dmcn.15044

16. Hua CL, Thomas KS, Bunker JN, et al. Dementia diagnosis in the hospital and outcomes among patients with advanced dementia documented in the Minimum Data Set. *J Am Geriatr Soc*. 2022;70(3):846-853. doi:10.1111/jgs.17564

17. Sattui SE, Navarro-Millan I, Xie F, Rajan M, Yun H, Curtis JR. Incidence of dementia in patients with rheumatoid arthritis and association with disease modifying anti-rheumatic drugs – Analysis of a national claims database. *Semin Arthritis Rheum*. 2022;57. doi:10.1016/j.semarthrit.2022.152083

18. Sullivan DR, Kim H, Gozalo PL, Bunker J, Teno JM. Trends in Noninvasive and Invasive Mechanical Ventilation among Medicare Beneficiaries at the End of Life. *JAMA Intern Med*. 2021;181(1):93-102. doi:10.1001/jamainternmed.2020.5640

19. Wang J, Monroe TB, Simning A, et al. Pain Management in Home Health Care: Relationship With Dementia and Facility Admissions. *Pain Management Nursing*. 2021;22(1):36-43. doi:10.1016/j.pmn.2020.06.007

20. Khalid S, Sambamoorthi U, Umer A, Lilly CL, Gross DK, Innes KE. Increased Odds of Incident Alzheimer's Disease and Related Dementias in Presence of Common Non-Cancer Chronic Pain Conditions in Appalachian Older Adults. *J Aging Health*. 2022;34(2):158-172. doi:10.1177/08982643211036219

© 2024 Gianattasio KZ et al. *JAMA Network Open.*
