# Supplementary material for: Case Definition for Diagnosed Alzheimer Disease and Related Dementias in Medicare
Source: JAMA Netw Open. 2024 Sep 3;7(9):e2427610. doi: 10.1001/jamanetworkopen.2024.27610 (PMC11372506; doi:10.1001/jamanetworkopen.2024.27610)
Supplement: Supplement 2. — Data Sharing Statement [file jamanetwopen-e2427610-s002.pdf]

Data Sharing Statement

Gianattasio. Case Definition for Diagnosed Alzheimer Disease and Related Dementias in Medicare. *JAMA Netw Open*. Published September 03, 2024.
doi:10.1001/jamanetworkopen.2024.27610

Data
Data available: No

Additional Information
Explanation for why data not available: We used Medicare Research Identifiable Files, which are not available for sharing publicly.
